# Tracking the longitudinal course of physiologic and mental health functioning among individuals in substance use disorder treatment

**DOI:** 10.3389/fpsyt.2026.1755153

**Published:** 2026-06-10

**Authors:** Wendy Insalaco, Charlotte Clapham, Brett Gelino, Jami Mayo Barney, Brianna Billings, Jennifer D. Ellis, J. Gregory Hobelmann, Andrew S. Huhn, Vadim Zipunnikov, Jill A. Rabinowitz

**Affiliations:** 1 Ashley Addiction Treatment, Havre de Grace, MD, United States; 2 Department of Biostatistics, Johns Hopkins University, Baltimore MD, United States; 3 Department of Psychiatry, Robert Wood Johnson Medical School, Piscataway, NJ, United States; 4 Department of Psychiatry and Behavioral Services, Johns Hopkins University, Baltimore, MD, United States

**Keywords:** Heart rate variability (HRV), photoplethysmography (PPG), recovery, resting heart rate (RHR), substance use disorder (SUD), wearable

## Abstract

**Introduction:**

Mental health monitoring is crucial to long-term recovery in substance use disorder (SUD) treatment; however, little is known about how changes in physiological indicators align with changes in self-reported mental health over time.

**Methods:**

We examined longitudinal associations of resting heart rate (RHR) and heart rate variability (HRV) collected via a WHOOP® photoplethysmography device with self-reported stress, anxiety, and depressive symptoms among individuals in SUD treatment. Participants (*N* = 59) continuously wore the device and completed at least two mental health and stress assessments during the first month of residential treatment.

**Results:**

Linear regression results indicated favorable changes in mental health and/or physiologic metrics, with notable heterogeneity in concurrent subject-level trends. Among participants with decreased RHR (better physiological functioning), 39% (*N*=23) also endorsed decreased stress, 42% (*N*=25) decreased anxiety, and 39% (*N*=23) improved depressive symptoms. Of those with increased HRV (greater stress adaptability), 39% (*N*=23) endorsed decreased stress, 39% (*N*=23) improved anxiety, and 41% (*N*=24) reduced depressive symptoms.

**Discussion:**

Concurrent changes in physiologic and mental health metrics during the first month of treatment varied across participants. These findings highlight the importance of integrating subjective mental health measures with physiological indicators to capture clinically relevant change during early SUD treatment.

## Introduction

In 2023, more than 4.5 million adults with mental illness sought treatment for substance use disorder (SUD) in the United States (1). Clinically significant anxiety (94.5%) and depression (89.6%) are common comorbidities observed in individuals in SUD treatment (2). These co-occurring symptoms are associated with a host of negative outcomes, including persistent psychological distress, greater likelihood of suicidal ideation and attempts, persistent substance-related craving, greater risk of overdose, challenges with treatment engagement, and an increased likelihood of treatment dropout (2, 3). Although many individuals in SUD treatment experience a reduction in anxiety and depression symptoms, others continue to struggle with persistent or worsening symptoms (4, 5). Thus, there is a need to more clearly identify individual-specific factors influencing these symptom trajectories to inform targeted mental health interventions and maximize SUD recovery.

Physiologic measures, including resting heart rate (RHR) and heart rate variability (HRV), have been linked to mental health outcomes in adults, including perceived stress, anxiety, and depressive symptoms (6, 7). Indeed, in adults, there is strong evidence linking depression- and anxiety-like symptoms to disrupted resting heart rate (RHR)—the number of heart beats per minute measured during an inactive, resting state—and heart rate variability (HRV)— a measure of the time variation between each heartbeat that reflects the balance between the sympathetic and parasympathetic nervous systems (8–10). In healthy adults, RHR typically ranges between 60–100 bpm with a mean of 79.1 bpm (11), while HRV measured by the root mean square of successive differences between heartbeats commonly falls between approximately 30–70 ms (12). In clinical samples meeting diagnostic criteria for depression, elevated RHR has been associated with increased anxiety and depressive symptoms (9). When compared with healthy controls, individuals meeting diagnostic criteria for depression (10, 13, 14) and anxiety disorders (7) exhibited lower HRV. HRV has also been significantly inversely related to depressive (10, 13) and anxiety (7) symptom severity in clinical samples. While a recent systematic review reported that RHR and HRV have been linked to anxiety and depressive symptom severity among clinical populations (Ramesh et al., 2023), few studies have specifically focused on individuals with SUDs (8, 15).

Some studies suggest that HRV and RHR may be useful physiologic measures for monitoring mental health symptoms in individuals undergoing treatment for SUDs (15, 16); however, direct evidence linking RHR and HRV to anxiety and depression within this population remains limited (16, 17), and, to our knowledge, no studies have examined longitudinal changes in these measures. There is some evidence that individuals with SUD experience lower HRV (15, 18), potentially indicative of poorer self-regulation and challenges in responding to environmental demands (6, 19). Given current interest in developing predictive models for SUD rehabilitation using digital biomarkers and psychological assessments (20–22) and recent research showing high comorbidity of anxiety and depression with SUD (2), further investigation is warranted. Because individuals reporting depression-like symptoms have disproportionately greater odds of early termination of SUD treatment (5), the interrelation of HRV and mental health across treatment offers a compelling focus for further investigation (23, 24).

RHR and HRV can be assessed via wearables that use photoplethysmography (PPG) technology to measure blood flow changes through optical sensors. Wearable devices that can assess physiologic measures in this manner are considered low burden and non-invasive for participants (25). Although these devices have already been implemented in some naturalistic (26, 27) and clinical settings, including evaluation of anxiety symptom severity (7), they have seldomly been used to track measures of physiological functioning among individuals in SUD treatment.

The allostatic model of addiction explains that chronic substance use produces dysregulation of stress and reward systems, resulting in a maladaptive physiological set point characterized by elevated sympathetic activity and reduced parasympathetic tone (28). This autonomic imbalance is reflected in reduced HRV and elevated RHR, biomarkers that have been associated with increased anxiety, depression, and impaired stress regulation (7, 9, 10, 13, 29). To better inform targeted intervention efforts for individuals in SUD treatment, there is a need to link real-time monitoring of changes over time in RHR, HRV, and clinically-relevant phenotypes that have been linked to critical SUD treatment outcomes. We hypothesize that during SUD treatment, gradual restoration of autonomic balance is expected, reflected in decreasing anxiety, depression, stress, and RHR and increasing HRV over time. The current study aimed to examine whether changes in RHR and HRV (assessed via the wearable WHOOP^®^) were associated with the longitudinal course of self-reported stress, anxiety, and depressive symptoms among patients in residential SUD treatment. The examination of concurrent trajectories of physiologic and mental health indicators during SUD treatment may inform tailored interventions and treatment planning.

## Materials and methods

### Participants

Longitudinal monitoring of patients during residential treatment was chosen for this study because the structured and substance-free environment of residential care provided relative stability of key behavioral and environmental factors, such as substance use, sleep, and daily routine, that may influence physiologic function. Participants (*N* = 169) were recruited from a 28-day residential program at Ashley Addiction Treatment to wear a PPG device throughout their stay and to complete weekly self-reported assessments. Patient self-reported data were collected via Trac9^©^, a third-party data collection provider that assesses and monitors patient outcomes. To encourage complete data collection, patients were registered in the Trac9^©^ platform at admission and asked to complete a baseline assessment within 24–48 hours during structured intake programming. Follow-up assessments were scheduled weekly and integrated into routine clinical programming. The system generated reminders for clinicians when surveys were overdue, and clinical and research staff periodically reminded participants to complete them.

During the first week of treatment, participants were also fitted with a WHOOP^®^ wearable PPG device to measure cardiovascular outcomes during treatment. Participants were instructed to wear the device continuously, and a web-based platform was used to monitor connectivity in real-time. Research staff met with patients weekly to ensure proper syncing and connectivity. Both data collection methods were part of quality improvement initiatives within the treatment facility.

Despite these procedures, some missing data occurred due to factors inherent to the naturalistic residential treatment setting. Because patients have limited access to smartphones, the study used a beta Bluetooth proxy system that uploaded wearable data through campus routers rather than personal devices, which occasionally produced connectivity errors outside the study team’s control. Additionally, some patients entered treatment later in the study implementation period, completed fewer assessments due to scheduling demands, or discharged early. As this initiative was implemented as part of routine clinical care rather than a controlled research protocol, assessment completion was encouraged but not mandatory. Deidentified data was transferred to the study team for analyzes. To ensure analytic validity, analyzes were limited to participants with at least two valid self-report measurements. All analyzes reported here were acknowledged by the Johns Hopkins University School of Medicine Institutional Review Board.

Of the 169 individuals in the study, 75 had missing demographic information. Furthermore, 110 participants had fewer than two Trac9^©^ measurements recorded throughout the study period or more than two Trac9^©^ measurements taken on the same day. These 110 participants were excluded from the analyzes, yielding a final analytic sample of 59 participants.

### Heart rate measurements

Heart rate measurements were collected using a PPG device WHOOP^®^ worn by participants. The wrist strap used PPG to detect cardiac blood flow and rhythm via LED-emitted light. RHR was calculated from PPG measurement of pulse intervals, while HRV was derived from the root mean square of successive pulse intervals continuously measured during overnight sleep (30). WHOOP^®^ PPG has demonstrated acceptable agreement with electrocardiogram-derived RHR measures (30, 31) and day-to-day HRV measurement comparable to that of other devices used in biometric research protocols (31).

### Anxiety symptoms

Participants recorded their anxiety symptoms using the 16-item Penn State Worry Questionnaire (PWSQ; 32). The measure is completed via a 5-point Likert scale ranging from 1 (*not at all typical of me*) to 5 (*very typical of me*) (e.g. item, “My worries overwhelm me”). We created a sum score with higher scores reflecting higher levels of anxiety symptoms. The task has shown excellent internal consistency (e.g., >.90) and high test-retest reliability (e.g., >.70; 32).

### Depression severity

Participants reported on their depression symptom severity via the self-report Center for Epidemiological Studies of Depression (CES-D) scale (33). The 20-item measure of depression severity is completed using a 4-point Likert scale ranging from 0 (*rarely or none of the time*) to 3 (*most or all of the time*) (e.g. item, “I felt depressed”). We created a sum score with higher scores reflecting greater experiences of depressive symptoms. Psychometric evaluations indicate strong internal consistency and acceptable test-retest reliability (e.g., >.50; 33).

### Stress

Participants self-reported on their experience of stress using the 10-item Perceived Stress Scale (PSS-10), a widely used measure that evaluates how often individuals perceive their lives as unpredictable, uncontrollable, or overwhelming (34). The PSS-10 uses a 5-point Likert scale ranging from 0 (*never*) to 4 (*very often*) (e.g., “In the last month, how often have you felt things were going your way?”). We created a sum score with higher scores reflecting greater perceived stress. The task exhibits acceptable test-retest reliability (e.g., >.55; 34).

### Statistical analysis

Descriptive analyzes (e.g., correlations) were performed to visually assess distributions and the relationships between physiological metrics (i.e., RHR, HRV) and self-reported mental health (i.e., perceived stress, anxiety, and depression). Intensive longitudinal measurements are often summarized at the subject level using aggregation procedures that provide simple and interpretable summaries such as subject-level mean, standard deviation, autocorrelation, etc. (35). Our goal was to construct a similarly simple subject-level descriptor that captures directional change over the study period (i.e., increase vs decrease). We fit subject-level linear regressions to obtain an estimate of change over time. For each of the 59 individuals, five simple linear regressions were conducted— one for each of the five measured variables (anxiety, depression, stress, RHR, and HRV). This was done to determine subject-specific changes in anxiety, depression, stress, RHR, and HRV over time. The slope of each regression represents the average change in the outcome per additional day in the study. Importantly, to further mitigate uncertainty due to the small number of observations per participant, we did not rely on the magnitude or statistical significance of these slopes. Instead, we used only the direction (sign) of the estimated slope (increase vs decrease) as an interpretable summary of within-person change.

Within-person change was examined to evaluate patterns of concordance between the self-reported and physiological measures across individuals. Negative slopes corresponding to each of the three self-reported metrics (depression, stress, anxiety) generally denote positive changes in participants. For the two physiological measurements, a negative slope reflecting change over time in RHR generally denotes positive changes, whereas a positive slope corresponding to HRV is thought to represent improvement in this metric. For each variable, we report the average, standard deviation (SD) and median slope across all individuals. To determine the level of agreement in change between physiological indicators and self-report measures, we created a contingency table for each pair of self-reported and physiological metrics such that participants were categorized by their concurrent trend (increasing or decreasing) in self-reported versus the physiological measurements.

We conducted sensitivity analyzes to assess the stability of findings as a function of the number of participant-specific assessments available for the analyzes. We restricted the sample to participants with at least 3 assessments (n = 29), excluding those with only two assessments (n = 30). Individual-level slopes for RHR, HRV, anxiety, depression, and stress were summarized (mean, SD, median), and directional change was examined via cross-tabulations. All analyzes were conducted using RStudio version 4.4.0.

## Results

**Table 1** presents a comparison of baseline demographic and clinical characteristics between participants included in the analytic sample and excluded participants with available baseline data. No statistically significant between-group differences were observed for age (independent-samples *t*-test, *t* = -0.95, *p* = 0.35), sex (chi-square test, *χ*² = 0.25, *p* = 0.62), primary substance use (Fisher’s exact test, *p* = 0.49), primary mental health condition (Fisher’s exact test, *p* = 0.80), or primary medical condition (Fisher’s exact test, *p* = 0.53). Of the 59 participants in the final sample, the majority were male (*N* = 43, 73%) with an average age of 37 years (*SD* = 12.9). More than three-quarters of the participants reported alcohol as a primary substance (*N* = 48, 81%). Other substances were reported at markedly lower frequencies, with cannabis and cocaine each reported by three participants (5%), and opioids by two participants (3%). An additional three participants reported primarily using a substance such as hallucinogens, other stimulants, or sedatives.

**Table 1:** Baseline characteristics of included participants and excluded participants with available baseline data.

Characteristic	Included (*n* = 59)	Excluded (*n* = 35)	*P*-value
Age, years	37.0 ± 12.9	40.0 ± 15.4	0.35^a^
Sex, *n* (%)			0.62^b^
Male	43 (72.9)	23 (65.7)	
Female	16 (27.1)	12 (34.3)	
Primary substance use, *n* (%)			0.49^c^
Alcohol	48 (81.4)	23 (65.7)	
Opioids	2 (3.4)	3 (8.6)	
Cannabis	3 (5.1)	3 (8.6)	
Cocaine	3 (5.1)	3 (8.6)	
Other	3 (5.1)	3 (8.6)	
Primary mental health condition, *n* (%)			0.80^c^
Anxiety	18 (30.5)	9 (25.7)	
Depression/depressive episode	7 (11.9)	3 (8.6)	
ADHD	4 (6.8)	4 (11.4)	
Other	1 (1.7)	5 (14.3)	
None reported	25 (42.4)	14 (40.0)	
Primary medical condition, *n* (%)			0.53^c^
Essential hypertension	9 (15.3)	8 (22.8)	
Gastro-esophageal reflux disease	4 (6.8)	1 (2.9)	
Hyperlipidemia	3 (5.1)	0	
Other	10 (16.9)	4 (11.4)	
None reported	33 (55.9)	22 (62.9)	

Seventy-five excluded participants were missing all baseline demographic and clinical data and were therefore omitted from this comparison table. Values are mean ± standard deviation or n (%). P-values are shown for between-group comparisons only.

^a^ Independent-samples t-test.

^b^ Chi-square test of independence.

^c^ Fisher’s exact test.

**Table 2** depicts correlations among study metrics. Self-reported depression and stress were highly correlated (*r* = .75, *p* < 0.001), as was anxiety with depression (*r* = .56, *p* < 0.001) and stress (*r* = .55, *p* < 0.001). RHR and HRV were negatively correlated (*r* = -.47, *p* < 0.001). Results from the primary analyzes are presented below.

**Table 2:** Key correlations between self-reported mental health measures and physiological indicators.

Variable 1	Variable 2	Correlation (r)	*P*-value
Depression	Anxiety	0.56	<0.001
Depression	Stress	0.75	<0.001
Anxiety	Stress	0.55	<0.001
Resting Heart Rate	Heart Rate Variability	-0.47	<0.001

### Single metric trajectories

**Table 3** depicts the summaries of the 59 individual-level regressions per variable. Most of the analytic sample reported decreasing average stress (*N* = 39, 66%), with an average 0.02 unit decrease per day in the study (*SD* = 1.7, *Median* = -0.25). Although the average slope was close to 0, the median slope of -0.25 indicates that for half of these participants, each additional day in the study was associated with average stress reduced by 0.25 points or more. This suggests a small, but meaningful, decrease in stress.

**Table 3:** Summary of individual-level slopes of variables measured over the study course.

Variable	Slope over study time		
	Mean	SD	Median
Anxiety	-0.26	1.51	-0.30
Depression	-0.20	1.71	-0.43
Stress	-0.02	1.70	-0.25
Resting Heart Rate	-0.07	0.39	-0.05
Heart Rate Variability	0.16	0.84	0.09

Most participants also experienced decreasing average anxiety over the course of the study (*N* = 41, 69%). The average slope for reported anxiety during the study was -0.26 (*SD* = 1.5, *Median* = -0.3). This means that on average, each additional day in the study was associated with a 0.26-point decrease in average anxiety. Trends in average depression were similar such that the majority of participants evidenced decreases in average measures of depression (*N* = 38, 64%), with an average slope of -0.2 (*SD* = 1.7, *Median* = -0.4). This median slope of -0.4 indicates that for half of the participants, each additional day in the study was associated with average depression reduced by 0.4 points or more.

In terms of heart rate measurements, 64% (*N* = 38) of the analytic sample experienced decreasing average RHR. However, the average and median decrease in RHR associated with additional days in the study was close to 0 (*β* = -0.07, *SD* = 0.40, *Median* = -0.05). Sixty-one percent (*N* = 36) of the sample experienced increasing average HRV over the course of the study. The mean slope was 0.16, indicating that on average, each additional day in the study was associated with a 0.16-unit increase in HRV (*SD* = 0.84, *β* = 0.09).

### Concurrent metric trajectories

**Table 4** depicts concurrent changes in metrics over the study duration comparing physiological metrics against objective measures. Of the 59 participants, 13 participants experienced decreased average RHR and increased average anxiety, 16 participants demonstrated increased average RHR and decreased anxiety, and 25 participants (42%) evidenced both decreased RHR and decreased anxiety. Thus, 54 of the 59 participants (92%) manifested average improvement in at least one of the two measurements. Similarly, 53 (90%) of participants displayed improvements in either average RHR or depression (15 improved in RHR but not depression, 15 improved in depression but not RHR, and 23 improved in both metrics). Lastly, 54 (92%) of participants exhibited improvement in either average RHR or average stress, with 15 improving in RHR but not stress, 16 improving in stress but not RHR, and importantly, 23 (39%) showing improvement in both metrics.

**Table 4:** Cross-tabulation of changes in physiological measures and self-reported mental health measures over the study period.

Anxiety				
Physiological measure	Direction of change	Anxiety, decrease *n*	Anxiety, increase *n*	Total *n*
RHR	Decrease	25	13	38
RHR	Increase	16	5	21
HRV	Decrease	18	5	23
HRV	Increase	23	13	36
*Total*		41	18	*N* = 59
Depression				
Physiological measure	Direction of change	Depression, decrease *n*	Depression, increase *n*	Total *n*
RHR	Decrease	23	15	38
RHR	Increase	15	6	21
HRV	Decrease	14	9	23
HRV	Increase	24	12	36
*Total*		38	21	*N* = 59
Stress				
Physiological measure	Direction of change	Stress, decrease *n*	Stress, increase n	Total *n*
RHR	Decrease	23	15	38
RHR	Increase	16	5	21
HRV	Decrease	16	7	23
HRV	Increase	23	13	36
*Total*		39	20	*N* = 59

Values are frequencies. RHR, resting heart rate; HRV, heart rate variability. Decrease and increase indicate the direction of each participant’s individual slope over the study period. Totals are shown for each physiological measure and each mental health measure.

With respect to HRV, of the 59 participants, 13 presented increased average HRV but also increased average anxiety, 18 experienced decreased anxiety but decreased HRV, and 23 (40%) manifested the most favorable outcome-- decreased anxiety and increased HRV. This resulted in a total of 54 participants (92%) improving in at least one of the two metrics. Similarly, 50 participants (85%) improved in average HRV, depression, or both metrics (14 improved in depression but not in HRV, 12 improved in HRV but not in depression, and 24 improved both in depression and in HRV metrics). Finally, the majority of the analytic sample also improved in either average HRV or patient-reported stress. 16 participants improved in stress but not in HRV, 13 participants improved in HRV but not in stress, and 23 improved in both metrics, resulting in 52 participants (88%) improving in at least one metric.

### Sensitivity analyzes

Consistent with results from the primary analyzes, anxiety (*M* = -0.41), depression (*M* = -0.46), stress (*M* = -0.35) and RHR (*M* = -0.15) decreased over time, while HRV increased (*M* = 0.11). Slopes were modestly more extreme and less variable, with similar medians, indicating stable central tendencies. Directional patterns were also consistent (i.e. decreases in RHR and increases in HRV generally aligned with improvements in psychological outcomes, with some discordance). See **Supplementary Tables 1- S2**.

## Discussion

This study examined changes in both physiological regulation—measured via RHR and HRV—and self-reported clinically relevant phenotypes (i.e., stress, anxiety, and depression) over the course of residential substance use disorder treatment. To our knowledge, no studies have simultaneously tracked and examined changes in these domains longitudinally at high resolution within a residential treatment setting or modeled these changes at the individual level. Results revealed that most participants exhibited meaningful improvements in at least one domain, although patterns of change strongly varied across individuals. As such, these findings challenge the adequacy of relying solely on either subjective reports or physiological measures when assessing recovery progress. A multimodal approach may more accurately capture clinically relevant change, particularly given the substantial heterogeneity in response trajectories observed here.

Prior work leveraging wearable-derived cardiac metrics in substance use contexts has generally conducted high-resolution data extraction and time-linked modeling of behavior. For example, Zhao et al. (36) derive HR from longitudinal data and apply a five-step analytical process to identify features temporally associated with vaping episodes. This framework prioritizes within-person, event-level sensitivity and classification-oriented inference. Similarly, Eddie, Wieman et al. (2023) (37) examine HRV dynamics during early recovery from alcohol use disorder using repeated assessments across treatment, modeling trajectories of autonomic regulation in relation to recovery processes and clinical outcomes.

In contrast, the present study adopts a deliberately parsimonious analytic strategy centered on subject-level linear regressions to estimate the directionality of change in cardiac indices across time. Rather than modeling fine-grained temporal associations or extracting high-dimensional feature sets, we reduce each individual’s trajectory to a single slope parameter and classify it by sign (i.e., increase vs. decrease). This approach shifts the inferential focus from prediction or time-localized association to coarser-grained characterization of physiological change.

This distinction is intentional and clinically motivated. In treatment settings where data completeness and signal quality are variable, and where the primary question concerns overall recovery-related change rather than momentary detection of substance use, the direction of univariate trends may be more interpretable and actionable than precise slope magnitude. By collapsing trajectories into directional indicators, the approach also facilitates estimation of the proportion of individuals demonstrating improvement versus deterioration over the treatment period.

Importantly, this approach is complementary to existing longitudinal wearable methodologies rather than a substitute for them. Feature-rich, time-resolved models, as in Zhao et al. (36), are well-suited for detecting proximal physiological signatures in substance use, while trajectory-based HRV analyzes, such as Eddie, Wieman et al. (2023) (37), characterize continuous change and its association with clinical endpoints. The slope-sign framework contributes an additional, low-dimensional lens that emphasizes interpretability and applicability in real-world clinical datasets.

Examination of univariate trends suggests a general improvement in self-reported mental health symptoms and/or cardiovascular indicators of physiologic regulation. At least half of the participants reported reduced average levels of stress, anxiety, and/or depressive symptoms. These results align with prior work documenting secondary mental health benefits of substance use treatment, including reductions in self-reported anxiety and depression (e.g., 38). Most participants also appeared to improve in physiologic regulation throughout treatment. This is a notable advancement in the understanding of how cardiovascular regulation changes during treatment, as prior studies have largely focused on acute changes during withdrawal or early stabilization (e.g., 39).

Importantly, not all participants reported improvements in these domains. We observed notable heterogeneity in how the concurrent change in these measures emerged in our sample. For example, in discrete comparisons across study variables, only about one-third of participants experienced joint improvements in both respective mental health and physiological measures, with the remaining participants worsening in at least one domain. Thus far, the broader literature has documented only concurrent changes in these metrics (e.g., 40). Indeed, some have pointed to these cardiovascular measures—in particular HRV—as plausible biomarkers or risk factors for adverse mental health outcomes (see reviews by 9, 41, 42). Our findings instead suggest that, among residents of an inpatient SUD treatment program, the relations may be inconsistent such that change in PPG-measured cardiac indicators of (physiological) stress may not reliably predict subsequent change in mental health across individuals in this setting.

This heterogeneity may offer crucial insight for predicting treatment outcomes, as research suggests that changes in cardiovascular and emotion regulation predict abstinence after treatment (37). Rather than assuming a uniform treatment effect, these findings suggest the need to identify distinct subgroups with diverging recovery trajectories—an approach that could inform more precise and effective treatment matching or augmentation strategies. In the meantime, clinicians and researchers should consider regularly monitoring these variables during treatment. Doing so may offer clinicians early insight into recovery trajectories and flag patients at risk of suboptimal outcomes—particularly when changes in objective and subjective markers diverge.

In summary, these data have notable clinical implications with potential to advance SUD treatment protocol. Calls for greater integration of psychological care during SUD treatment are well supported (e.g., 43, 44), yet few studies have tracked changes in both emotional dysregulation and physiological regulation concurrently. Further, despite the widespread adoption of wearables by the public and their potential to complement treatment planning efforts, their implementation and use in SUD treatment settings remains limited. Facilities that do not currently engage wearable monitoring technologies can still meaningfully assess physiologic recovery through structured symptom measurement, behavioral indicators of stress regulation, and clinical observation. These approaches provide practical alternatives for monitoring treatment progress and may serve as foundational steps toward integrating biometric assessment into routine care. Future research should examine whether improvements in cardiovascular regulation are causally or independently linked to improvements in mental health, as both may reflect a common underlying mechanism (e.g., autonomic balance). Findings underscore the translational potential of integrating passive physiological monitoring into routine residential treatment. Thus, more research is also needed to identify whether there are latent classes (e.g., those with initial symptom severity, differing baseline HRV levels, or distinct comorbid profiles) among the portion of individuals not reporting concurrent improvements in cardiovascular and mental health.

## Strengths and limitations

This study made use of continuous psychophysiological data collection in the form of a PPG device worn by participants for the duration of residential treatment. This offered fine-grained and objective data collection and monitoring of RHR and HRV—a significant strength of this study. Paired with regular self-report assessments, this dataset enabled nuanced exploration of within-person change across multiple domains of recovery. That said, in roughly half of the participants (*N* = 30, 51%), the self-report measures of anxiety and depressive symptoms were limited to only two measurements, restricting our ability to detect finer-grained or dynamic changes in emotional states across the course of treatment. A key limitation of this study is that reducing trajectories to a linear slope and its direction may obscure non-linear patterns, phase-specific dynamics, or short-term fluctuations that more granular longitudinal models are designed to detect. As such, the present method is best viewed as a pragmatic summary metric that complements, rather than replaces, more complex time-series analyzes.

Future work might implement a more vigorous psychological measure to achieve a more comprehensive and longitudinal depiction of mood states and internalizing symptoms. In addition, future research might examine several factors beyond the scope of the current study, including the effects of acute withdrawal, which may confound physiological measures independent of psychological state; the possibility that physiological and psychological recovery operate through distinct, non-overlapping mechanisms; and the potential role of medication-assisted treatment in modulating cardiovascular measures independently of mental health.

## Conclusions

This study offers novel insight into the multidimensional recovery process experienced among patients in residential substance use treatment. We jointly tracked within-person changes in both physiological and psychological functioning. Findings indicate that most participants improved in at least one domain during treatment—either cardiovascular regulation or emotional wellbeing—with some improving in both. These results not only support the efficacy of residential treatment for addressing co-occurring dysregulation, but also emphasize the importance of individualized, multimodal assessment strategies. While observational, this study lays important groundwork for future mechanistic work exploring how cardiovascular and psychological regulation interact to influence long-term recovery outcomes. As research continues to explore predictors of long-term recovery, future work should examine how these within-person changes relate to treatment retention, post-discharge outcomes, and opportunities for targeted intervention in real time.

## Data Availability

The raw data supporting the conclusions of this article will be made available by the authors, without undue reservation.
