# Supplementary material for: Tracking the longitudinal course of physiologic and mental health functioning among individuals in substance use disorder treatment
Source: Front Psychiatry. 2026 Jun 10;17:1755153. doi: 10.3389/fpsyt.2026.1755153 (PMC13290847; doi:10.3389/fpsyt.2026.1755153)
Supplement: Supplementary file 1 [file DataSheet1.pdf]

Supplementary Table S1. Summary of individual-level slopes of variables measured over the study course

Variable 1	Slope over study time		
	Mean	SD	Median
Anxiety	-0.41	0.66	-0.30
Depression	-0.46	0.71	-0.44
Stress	-0.25	0.46	-0.28
Resting Heart Rate	-0.15	0.25	-0.11
Heart Rate Variability	0.11	0.48	0.07

Supplementary Table S2. Cross-tabulation of changes in physiological measures and self-reported mental health measures over the study period

Anxiety				
**Physiological Measure**	**Direction of Change**	**Anxiety, Decrease *n***	**Anxiety, Increase *n***	**Total *n***
RHR	Decrease	15	7	22
RHR	Increase	6	1	7
HRV	Decrease	10	1	11
HRV	Increase	11	7	18
Total		**21**	**8**	***N* = 29**

Depression				
**Physiological Measure**	**Direction of Change**	**Depression, Decrease *n***	**Depression, Increase *n***	**Total *n***
RHR	Decrease	15	7	22
RHR	Increase	6	1	7
HRV	Decrease	8	3	11
HRV	Increase	13	5	18
Total		**21**	**8**	***N* = 29**

Stress				
**Physiological Measure**	**Direction of Change**	**Stress, Decrease *n***	**Stress, Increase *n***	**Total *n***
RHR	Decrease	16	6	22
RHR	Increase	7	0	7
HRV	Decrease	9	2	11
HRV	Increase	14	4	18
Total		**23**	**6**	***N* = 29**
